# Risk factors for low birth weight in Bale zone hospitals, South-East Ethiopia : a case–control study

**DOI:** 10.1186/s12884-015-0677-y

**Published:** 2015-10-13

**Authors:** Habtamu Demelash, Achenif Motbainor, Dabere Nigatu, Ketema Gashaw, Addisu Melese

**Affiliations:** Department of Public Health, College of Medicine and Health Sciences, Madawalabu University, Bale, Goba, Ethiopia; Department of Nursing College of Medicine and Health Sciences, Madawalabu University, Bale, Goba, Ethiopia; Department of Medicine College of Medicine and Health Science, Madawalabu University, Bale, Goba, Ethiopia

**Keywords:** Maternal risk factors, Low birth weight, Environmental risk factors, Socio economic risk factors

## Abstract

**Background:**

Low birth weight (LBW) is closely associated with foetal and neonatal mortality and morbidity, inhibite growth and cognitive development and resulted chronic diseases later in life. Many factors affect foetal growth and thus, the birth weight. These factors operate to various extents in different environments and cultures. The prevalence of low birth weight in the study area is the highest in the country. To the investigator’s knowledge in Bale Zone, no study has yet been done to elucidate the risk factors for low birth weight using case control study design. This study was aimed to identify the risk factors of low birth weight in Bale zone hospitals.

**Methods:**

A case–control study design was applied from April 1st to August 30th, 2013. A total of 387 mothers (136 cases and 272 controls) were interviewed using structured and pretested questionnaire by trained data collectors working in delivery ward. For each case, two consecutive controls were included in the study. All cases and controls were mothers with singleton birth, full term babies, no diabetes mellitus and no hypertensive. The data were entered and analyzed using SPSS version 16.0 statistical package. The association between the independent variables and dependent variable (birth weight) was evaluated through bivariate and multiple logistic regression analyses.

**Result:**

Maternal age at delivery <20 years (adjusted odds ratio (AOR) = 3; 95 % confidence interval (CI) = 1.65–5.73), monthly income <26 United States Dollarr (USD) (AOR = 3.8; 95 % CI = 1.54–9.41), lack of formal education (AOR = 6; 95 % CI = 1.34–26.90), being merchant (AOR = 0.1; 95 %CI = 0.02–0.52) and residing in rural area (AOR = 2.1; 95 % CI = 1.04–4.33) were socio-economic variables associated with low birth weight. Maternal risk factors like occurrence of health problems during pregnancy (AOR = 6.3; 95 % CI = 2.75–14.48), maternal body mass index <18 kg/m2 (AOR = 6.7; 95 % CI = 1.21–37.14), maternal height <1.5m (AOR = 3.7; 95 % CI = 1.22–11.28), inter-pregnancy interval <2 years (AOR = 3; 95 % CI = 1.58–6.31], absence of antenatal care (OR = 2.9; 95 % CI = 1.23–6.94) and history of khat chewing (AOR = 6.4; 95 % CI = 2.42–17.10) and environmental factors such as using firewood for cooking (AOR = 2.7; 95 % CI = 1.01–7.17), using kerosene for cooking (AOR = 8.9; 95 % CI = 2.54–31.11), wash hands with water only (AOR = 2.2; 95 % CI = 1.30–3.90) and not having separate kitchen room (AOR = 2.6; 95 % CI = 1.36–4.85) were associated with low birth weight.

**Conclusion:**

Women who residing in rural area, faced health problems during current pregnancy, had no antenatal care follow-up and use firewood as energy source were found to be more likely to give low birth weight babies. Improving a mother’s awareness and practice for a healthy pregnancy needs to be emphasized to reverse LBW related problems.

## Background

Low birth weight (LBW) is considered as the single most important predictor of infant mortality, especially of deaths within the first month of life [1]. It is a significant determinant of infant and childhood morbidity, particularly of neurodevelopmental impairments such as mental retardation and learning disabilities. It is also closely associated with foetal and neonatal mortality and morbidity, inhibite growth and cognitive development and chronic diseases later in life [2].

More than 20 million infants worldwide, representing 16 % of all births are born with low birth weight. The level of low birth weight in low income countries is more than double the level in middle income countries. About 10 % of births in Oceania were low birth weight births [3, 4]. The result of the 2005/6 demography and health survey report of Zimbabwe showed that the prevalence of low birth weight was 16 % and the prevalence varies across sex (17 % among females versus 13 % among males) [5].

The magnitude of LBW births are probably underestimates of the global situation because in the developing world a significant proportion of infants are born at home and not registered as live births [3]. According to the 2005 Ethiopian demography and health survey, 14 % of babies in Ethiopia were low birth weight [6]. After 5 years the prevalence decreased by 3 % and it was 11 % in 2011 [7]. The 2011 health and health related indicator in Ethiopia showed that the proportion of low birth weight in Oromia region was 28 % followed by Gambella region which was 26 % [8]. Based on the 2011 Ethiopian demography and health survey, Only 33.3 % of the gambela women received professional antenatal care service from health service institution and only 13.2 % of the gambela mother delivered at health care facility [7]. In Oromia region, only 3.7 % of women delivered at a professional health care facility. Over 95 % delivered at home with all the attendant risks and complications assisted only by traditional birth attendants and only aquarter of Oromia women (24.8 %) had received antenatal care from a professional care provider [8].

Ethiopia is known to be among countries with very high maternal and child mortality rate. Even though sufficient specific data on Oromiya and Gambela are lacking, It would not be a stretch to assume that the grim statistics would apply to the women in the two regions too [7].

Many factors determine the duration of gestation and foetal growth, and thus, the birth weight. They might be related to the infant, the mother, or the physical environment and play an important role in determining the birth weight and the future health of the infant [3]. In different parts of the world Studies showed that several LBW risk factors contribute for the presence of the problem. Hypertension, weight gain during pregnancy, body size (mainly maternal pre pregnancy weight) and low social class were some of from others [9].

Birth weight is affected to a great extent by the mother’s own foetal growth and her diet from birth to pregnancy. Mother’s poor nutrition and health, high prevalence of specific and non-specific infections, pregnancy complications, and physically demanding work during pregnancy are contributes to poor foetal growth [3].

In order to prevent LBW, its main modifiable risk factors need to be understood. Additionally, the interrelationships between maternal, social and cultural factors need to be investigated. Results of the research would be critical to develop interventions aimed at modifying behaviors and other risk factors for low birth weight. Hence, this research was aimed to identifying the socio-economic, maternal and environmental risk factors for low birth weight in the study area to design urgent and sustainable interventions.

## Methods

### Study setting and population

A hospital based case control study was conducted in Bale zone from April 1^st^ to August 30^th^, 2013. Bale zone is the second largest zone in Oromia regional state located in the South-eastern part of Ethiopia. The zone administratively divided in to 17 districts and 6 town administration [Bale Zone administrative office 2013]. Based on bale zone health office report, there are four government hospitals (Goba, Robe, Ginnir and Delomena hospitals) and 76 functional health centers, 351 functional health post, 179 private clinic, 1 NGO clinic, 4 other public clinic, 95 pharmacy/drug shop, 1 NGO drug shop and 4 medical drug store in Bale zone.

All mothers who gave birth in the four governmental hospitals were the source population. Mothers who gave live births weighed less than 2500 g were considered as cases and live births weighed 2500 g and above as controls. Mothers who had diabetes mellitus, hypertension, preterm baby and multiple births were excluded; because those conditions are known risk factors for low birth weight.

### Sample size and sampling techniques

The sample size was determined using the proportion difference approach with the assumption of 95 % confidence level (Z_α/2_ = 1.96), 80 % power (Z_β_ = 0.84), control to case ratio 1:2 (r = 2), the odds ratio to be detected ≥ 2 and the 20 % control group will be exposed. The final sample size was 408 (136 cases and 272 controls).

The weight of all live births delivered in the four hospitals during the study period was measured. Based on the case definition those mothers who gave live births weighed less than 2500g included in the study as cases. For each case, two consecutive controls were included.

### Data collection procedure

Data was collected through face to face interview using structured and pretested questionnaire. The questionnaire was adopted from Ethiopian health and demographic survey (EDHS) and behavioral surveillance survey (BSS) and other peer reviewed articles [3, 6, 7]. The questionnaire included three sections. The first section of the questionnaire was related to socio demographic background. Information obtained from this section is important because the presence of economic deprivation has its own influence on the birth outcome of pregnant women. The second section included questionnaire which helps to assess the maternal condition like birth interval, number of children, maternal follow up and health problems as a cause of LBW. Questions in the third section were related to household environmental conditions like, source of water, source of energy, personal hygiene and number of individuals in the home. Information from this section has great implication on the birth outcome. Insufficient and unsafe water for pregnant women contributes infection which leads to low birth weight. The interview and anthropometric measurements were conducted by trained midwives and nurses working in labour ward.

The weight of the newborns was measured within 15 min after birth using a balanced Seca scale. The scale was always checked and zeroed before weighing each newborn. Maternal height was measured against a wall height scale to the nearest centimeter. Maternal weight was measured by beam balance to the nearest kilogram and body mass index (BMI) was subsequently calculated.

### Operational definition

#### Birth weight

The first weight of the new-borns measured within 15 min after birth. Low birth weight (cases) were those newborns weighed less than 2500g while those newborns with birth weight of 2500g and above were considered as normal weight (controls).

#### Preterm birth

It is a birth before a gestational age of 37 complete weeks.

#### Multiple births

It refers when more than one fetus is carried to term in a single pregnancy.

### Data processing and statistical analysis

First the data were checked for completeness and inconsistencies. Then coded and entered to SPSS version 16.0 soft ware. The entered data were cleaned and edited before subsequent analysis. Summary statistics such as mean and standard deviation was computed for cases and controls groups. The socio-demographic characteristics of the mothers were cross tabulated among cases and controls. Bivariate and multiple logistic regression analyses were done to identify the relationship between the independent variables (socio-economic, maternal and environmental factors) and dependent variable (birth weight).

The socio-economic factors; maternal age, residence, marital status, maternal education, maternal occupation, husband’s occupation, husband’s education, monthly income and role of decision making on money how to be used were entered to the bivariate model with low birth weight. Similarly, maternal factors including; birth interval, gravida, antenatal care (ANC) follow-up, gestational age at first ANC visit, deworming during pregnancy, maternal height, maternal weight, maternal BMI, history of pregnancy related problems, history of alcohol drinking and khat chewing were entered in to the bivariate model. Likewise; environmental factors entered to bivariate analysis were latrine availability, average daily household water consumption, mothers’ hand washing practice, availability of separate kitchen room, source of drinking water, solid waste disposal site and water source point accessibility to household.

The three sets of independent variables (socio-economic, maternal and environmental factors) that showed significant association in the bivariate logistic regression analysis were entered in multiple logistic regressions analysis using backward stepwise method. All statistical tests were two sided and significant association was declared at p-value less than 0.05.

Ethical clearance letter was obtained from research review committee (ERC) of Madawalabu University. Permission letters were secured from Bale Zone Health Bureau and from the four respective hospitals. Verbal consent was obtained from each mother prior to interview. Additionally, all the information obtained from each study participant was kept confidential throughout the process of this study.

## Results

From a total of 408 sample size, 387 mothers of (129 cases and 258 controls) were included in the interviewe which made the response rate of 94 % for both cases and controls.

### Socio economic and maternal characteristics

Almost half of mothers of the cases 51.2 % and more than two third of mothers of controls 69.4 % were in the age group of 21–35 years. About sixty-seven percent of mothers among cases and 53.9 % of mothers among controls were Muslim in religion. Larger proportions, 69.8 % of cases of mothers and 45.3 % of the controls mothers were housewives. About forty-six percent of mothers of LBW babies were illiterate while 15.5 % of mothers of normal birth weight (NBW) babies were illiterate. Concerning monthly family income, relatively higher percentage of mothers of low birth weight babies 24.6 % had an income less than 26$ ompared to mothers of normal birth weight babies 7.8 % [Table 1].

**Table 1:** Distribution of mothers by socio demographic characteristics in Bale Zone, South east Ethiopia, August, 2013

Variables	LBW		NBW		Total	
Age group (Years )	No	%	No	%	No	%
≤ 20	52	40.3	56	21.7	108	28.0
21–35	66	51.2	179	69.4	245	63.3
> 35	11	8.5	23	8.9	34	8.7
Weight group (Kg)						
< 50	16	12.4	12	4.7	28	7.2
≥ 50	113	87.6	246	95.3	359	92.8
Height (Cm)						
≤ 150	20	15.5	16	6.2	36	9.3
> 150	109	84.5	242	93.8	351	90.7
Birth interval in year						
≤ 2	32	49.2	43	25.3	75	31.9
> 2	33	50.8	127	74.7	160	68.1
Religion						
Muslim	86	66.7	139	53.9	225	58.1
Orthodox	38	29.5	100	38.8	138	35.7
Protestant	5	3.9	14	5.4	19	4.9
Catholic	0	0	5	1.9	5	1.3
Ethnicity						
Oromo	103	79.8	183	70.9	186	73.9
Amhara	14	10.9	64	24.8	78	20.2
Other^b^	12	9.3	11	1.3	23	5.9
Marital status						
Married	116	89.9	243	94.2	359	92.7
Others^a^	13	10.1	15	5.8	28	7.3
Residence						
Urban	67	51.9	194	75.2	261	67.4
Rural	62	48.1	64	24.8	126	32.6
Head of household						
Male	117	90.7	233	90.3	350	90.4
Female	12	9.3	25	9.7	37	9.6
Maternal Occupation						
Employed	10	7.8	40	15.5	50	12.9
House wife	90	69.8	117	45.3	207	53.4
Farmer	8	6.2	31	12.0	39	10.0
Merchant	12	9.3	63	24.4	75	19.3
Daily labourer	9	7.0	7	2.7	16	4.3
Monthly Income (USD^c^)						
< =26	31	24.6	20	7.8	51	13.3
27–53	40	31.7	49	19.1	89	23.2
54–79	15	11.9	39	15.2	54	14.1
> 79	40	31.7	149	58	189	49.3
Maternal Education						
Illiterate	59	45.7	40	15.5	99	25.6
Read and write only	2	1.6	5	1.9	7	1.8
Primary (1–8)	49	38.0	109	42.2	158	40.8
Secondary (9–12)	14	10.9	81	31.4	95	24.5
Tertiary	5	3.9	23	8.9	28	7.2

^a^Include: divorced, single, separated and widowed

^b^include: Tigrie, Guragie, Somali and Wolayita

^c^United States dollar

Nearly half of mothers with LBW babies 50.8 % spaced between present and past pregnancy more than 2 years compared to mothers with NBW babies 74.7 %. Seventy six percent of mothers among cases and 82 % of mothers among controls had BMI of 18.5–25 kg/m^2^. Maternal height, 84.5 % from cases and 93.8 % from controls were greater than 150cm tall. Among mothers of cases 48.1 % and mothers of controls 24.8 % lived in rural part of the study area and most of mothers 93 % were currently married [Table 1].

### Risk factors for low birth weight

Bivariate logistic regression analyses were performed between socio-economic factors of mothers and low birth weight. The analyses revealed that maternal age, residence, maternal education, maternal occupation, husband’s occupation, husband’s education, monthly income and participation on decision on how money be used were statistically significant with low birth weight in the bivariate model. Those socio economic factors of the mothers which have significant association with low birth weight in the bivariate model were entered to multiple logistic regression analyses. The results showed that mothers who were residing in rural areas were two times more prone to deliver LBW babies than their urban counterparts (AOR = 2.1; (95 % CI = 1.04–4.33)). Those mothers with monthly income less than 26$ were four times more likely to give LBW baby as compared to mothers with monthly income of greater than 79 $ (AOR = 3.8; (95 % CI = 1.54–9.41)). Mothers who had no formal education were at higher risk to give low birth weight baby as compared to mothers with tertiary level of education (AOR = 6; (95 % CI = 1.34–26.90)). Mothers who were in the age group of less than 20 years were more likely to deliver low birth weight babies than those mothers in the age group of 21–35 years (AOR = 3.1; (95 % CI = 1.65–5.73)). Mothers who were merchant by their occupational were 90 % less likely to deliver low birth weight babies compared to employed mothers (AOR = 0.1; (95 % CI = 0.02–0.52)) (Table 2).

**Table 2:** Associations between low birth weight (LBW) and socio economic factors among mothers in Bale Zone, Oromia regional state, August, 2013

Factors	LBW		NBW		Crude OR [95 % CI]	*p*-value	Adjusted OR [95 % CI]	*P*-Value
	No	%	No	%				
Maternal age (year)								
≤ 20	52	40.3	56	21.7	2.5 [1.57, 4.03]	<0.001	3.1 [1.65,5.73]	<0.001
21–35	66	51.2	179	69.4	1		1	
> 35	11	8.5	23	8.9	1.3 [0.59, 2.81]	0.509	0.9 [0.35, 2.71]	0.963
Residence								
Urban	67	51.9	194	75.2	1		1	
Rural	62	48.1	64	24.8	2.8 [1.80,4.38]	<0.001	2.1 [1.04,4.33]	0.040
Marital status								
Married	116	89.9	243	94.2	1			
Others	13	10.1	15	5.8	1.8 [0.84,3.94]	0.131		
Maternal Education								
No education	61	47.2	45	17.4	6.2 [2.20,17.66]	0.001	6.0 [1.34,26.90]	0.019
Primary (1–8)	49	38.0	109	42.2	2.1 [0.74,5.74]	0.164	1.6 [0.38,6.68]	0.523
Secondary (9–12)	14	10.9	81	31.4	0.8 [0.26, 2.44]	0.688	0.7 [0.19,2.88]	0.660
Tertiary	5	3.9	23	8.8	1		1	
Maternal occupation								
Employed	10	7.8	40	15.5	1		1	
House wife	90	69.8	117	45.3	3.1 [1.46, 6.48]	0.003	0.5 [0.15,1.87]	0.326
Farmer	8	6.2	31	12.0	1.0 [0.36, 2.92]	0.952	0.1 [0.03,0.58]	0.008
Merchant	12	9.3	63	24.4	0.8 [0.30,1.93]	0.566	0.1 [0.02,0.52]	0.005
Daily labourer	9	7.0	7	2.7	5.1 [1.54, 17.19]	0.008	0.68 [0.09,5.23]	0.713
Husband education								
No education	64	52.0	60	24.4	3.5 [1.79,6.72]	<0.001		
Primary (1–8)	27	22.0	50	20.3	1.8 [0.85,3.64]	0.131		
Secondary (9–12)	16	13.0	84	34.2	0.6 [0.29,1.34]	0.225		
Tertiary	16	13.0	52	21.1	1			
Husband occupation								
Employed	24	19.5	77	31.3	1		1	
Farmer	59	48.0	81	32.9	2.3 [1.32, 4.12]	0.003	0.5 [0.18,1.23]	0.125
Merchant	32	26.0	78	31.7	1.0 [0.71, 2.44]	0.382	1.6 [0.69,3.82]	0.265
Daily labourer	8	6.5	10	4.1	2.6 [0.91, 7.24]	0.075	1.2 [0.33,4.24]	0.804
Monthly income (USD)								
< =26	31	60.8	20	39.2	5.8 [2.98,11.19]	<0.001	3.8 [1.54,9.41]	0.004
27–53	40	31.7	49	19.1	3.0 [1.76,5.24]	<0.001	1.9 [0.93,3.89]	0.076
54–79	15	11.9	39	15.2	1.4 [0.72, 2.86]	0.307	1.3 [0.58,2.95]	0.520
> 79	40	31.7	149	58.0	1		1	
Decision on resource utilization								
Mother alone	13	39.4	20	60.6	1		1	
Jointly	81	26.6	224	73.4	0.6 [0.27, 1.17]	0.122	0.6 [0.15,2.56]	0.518
Husband only	35	71.4	14	28.6	3.8 [1.51,9.78]	0.005	2.2 [0.47,10.29]	0.315

Similarly, bivariate logistic regression analyses were done to check the presence of significant association between maternal factors and low birth weight. As a result; birth interval, gravida, ANC follow-up, gestational age at first ANC visit, maternal height, maternal weight, maternal BMI, history of pregnancy related problems, history of alcohol drinking and history of khat chewing were statistically associated with low birth weight. In multiple logistic regression analysis; mothers who encountered pregnancy related health problems during current pregnancy were at higher risk to deliver low birth weight baby than mothers who didn’t encounter any health problem (AOR = 6.3; (95 % CI = 2.75–14.48). The odds of low birth weight were higher among mothers who didn’t attend antenatal care for current pregnancy as compared to mothers who attended ANC (AOR = 2.9; (95 % CI =1.23–6.94). In the same manner; mothers with birth interval of 2 years and below between the current and previous birth were more likely to give low birth weight baby than mothers who gave birth greater than 2 years apart (AOR = 3.2; (95 % CI =1.58–6.31)). The odds of giving LBW baby were higher among mothers with body mass index (BMI) less than 18.50kg/m^2^ as compared to mothers with BMI greater than 25 kg/m^2^ (AOR = 6.7; 95 % CI = (1.21–37.14). Maternal short stature (≤150 cm) AOR = 3.7; 95 % CI = 1.22–11.28) and khat chewing (AOR = 6.4; 95 % CI =2.41–17.10) were risk factors for low birth weight [Table 3].

**Table 3:** Associations between low birth weight (LBW) and maternal risk factors among mothers in Bale Zone, South east Ethiopia, August, 2013

Factors	LBW		NBW		Crude OR (95 % CI)	*p*-value	Adjusted OR (95 % CI)	*P*-Value
	No	%	No	%				
ANC visit								
Yes	98	76.0	228	88.4	1		1	
No	31	24.0	30	11.6	2.4 [1.38,4.19]	0.002	2.9 [1.23, 6.94]	0.014
Gestational age at first ANC visit (months)								
< 4	11	11.2	33	14.5	1			
4–6	64	65.3	158	69.3	1.2 [0.58,2.55]	0.606		
> 6	23	23.5	37	16.2	1.9 [0.79,4.40]	0.155		
Maternal height (meter)								
≤ 1.50	20	15.5	16	6.2	2.8 [1.38,5.62]	0.004	3.7 [1.22,11.28]	0.021
> 1.50	109	84.5	242	93.8			1	
Maternal weight (Kg)								
< 50	16	12.4	12	4.7	2.9 [1.33,6.34]	0.007		
≥ 50	113	87.6	246	95.3	1			
Maternal BMI (Kg/m^2^)								
< 18.50	12	9.3	3	1.2	9.3 [2.34,36.6]	0.002	6.7 [1.21,37.14]	0.029
18.50–25.00	98	76.0	211	81.8	1.1 [0.60,1.94]	0.808	1.2 [0.51, 3.10]	0.617
> 25.00	19	14.7	44	17.1	1		1	
Gravida								
Primigravida	61	47.3	73	28.3	2.6 [1.62,4.10]	<0.001		
Multigravida	50	38.7	154	59.7	1			
Grandmultigravida	18	14.0	31	12.0	1.8 [0.92,3.47]	0.086		
Pregnancy Related health problem								
Yes	37	28.7	24	9.3	3.9 [2.22,6.92]	<0.001	6.3 [2.76,14.48]	<0.001
No	92	71.3	234	90.7	1		1	
Birth interval in year								
≤ 2	32	49.2	43	25.3	2.9 [1.58,5.20]	<0.001	3.2 [1.59,6.31]	0.001
> 2	33	50.8	127	74.7	1		1	
Deworming during pregnancy								
Yes	20	15.5	60	23.3	1			
No	109	84.5	198	76.7	1.7 [0.95,2.88]	0.078		
Ever Khat chewing								
Yes	30	23.3	18	7.0	4.0 [2.15,7.58]	<0.001	6.4 [2.42,17.10]	<0.001
No	99	76.7	240	93.0	1		1	
Ever drunk alcohol								
Yes	32	24.8	39	15.1	1.9 [1.10,3.13]	0.021		
No	97	75.2	219	84.9	1			

The household environmental factors including latrine availability, average daily household water consumption, and mothers’ hand washing practice, availability of separate kitchen room, solid waste disposal site and source of energy for cooking were statistically associated with LBW in the bivariate logistic regression analyses model. The multiple logistic regression results showed that; the likelihood of giving low birth weight baby was significantly higher among mothers who were used firewood for cooking than electricity (AOR = 2.7; (95 % CI = 1.00–7.17), kerosene than electricity (AOR = 8.9; (95 % CI = 2.53–31.11) and animal dung than electricity (AOR = 14.4; (95 % CI = 4.08–50.97)). Mothers from household which had no separate room for cooking significantly associated with low birth weight (AOR =2.5; (95 % CI = 1.35–6.40). Mothers who washed their hands with water only had higher probability of giving low birth weight baby than mothers who washed their hands using water with soap (AOR = 2.2; (95 % CI = 1.29–3.90) and hand washing with water and ash also found to be risky for low birth weight compared to using water with soap (AOR = 3.3; (95 % CI = 1.05–10.29). The odds of LBW babies among mothers with daily household water consumption less than 50 l were higher than mothers with daily household water consumption of 50 l and above (AOR = 1.8 ;( 95 % CI =1.02–3.21) [Table 4].

**Table 4:** Associations between low births weight (LBW) and Environmental factors among mothers in Bale Zone, South east Ethiopia, August, 2013

Factors	LBW		NBW		Crude OR (95 %CI)	p-value	Adjusted OR (95 % CI)	P-Value
	No	%	No	%				
Hand washing								
Water with Soap	77	59.7	211	81.8	1		1	
Water only	41	31.8	41	15.9	2.7 [1.65, 4.54]	<0.001	2.2 [1.30, 3.90]	0.004
Water with ash	11	8.5	6	2.3	5.0 [1.80,14.05]	0.002	3.3 [1.05,10.30]	0.041
Daily water consumption (L)								
≥ 50	37	28.7	112	43.4	1		1	
< 50	92	71.3	146	56.6	1.9 [1.21,3.00]	0.005	1.6 [0.99,2.76]	0.055
Source of drinking water								
Protected	118	91.5	233	90.3	1			
Unprotected	11	8.5	25	9.7	0.9 [0.41,1.83]	0.711		
Accessibility of water source								
Accessible	98	93.3	202	92.2	1			
Not accessible	7	6.7	17	7.8	0.8 [0.34,2.11]	0.725		
Solid waste disposal site								
Private pit	51	39.5	119	46.0	1			
Communal pit	33	25.6	84	32.6	0.9 [0.55,1.54]	0.743		
Used as fertilizer	9	7.0	16	6.2	1.3 [0.54,3.17]	0.545		
Burning	7	5.4	19	7.4	0.9 [0.34,2.17]	0.749		
Open dump	29	22.5	20	7.8	3.4 [1.75,6.53]	<0.001		
Separated kitchen room								
Yes	97	75.2	234	90.7	1		1	
No	32	24.8	24	9.3	3.2 [1.80,5.74]	<0.001	2.6 [1.36,4.85]	0.004
Sources of energy for cooking								
Electricity	5	3.9	41	15.9	1		1	
Fire Wood	82	63.6	190	73.6	3.5 [1.35,9.28]	0.010	2.7 [1.00,7.17]	0.049
Kerosene	13	10.1	11	4.3	9.7 [2.84, 33.07]	<0.001	8.9 [2.54,31.11]	<0.001
Animal dung	23	17.8	9	3.5	21.0 [6.27,70.03]	<0.001	14.4 [4.08,50.97]	<0.001
Others	6	4.7	7	2.7	7.0 [1.68, 29.43]	0.008	6.1 [1.40,26.27]	0.016
Availability of latrine								
Yes	112	86.8	246	95.3	1			
No	17	13.2	12	4.7	3.1 [1.44,6.73]	0.004		

## Discussion

Low birth weight can be influenced by various factors that occur prior to and during pregnancy including the household environmental conditions where the mothers live. Therefore; this study identified the risk factors for low birth weight which is important for proper, immediate and sustainable intervention to improve maternal health for better pregnancy outcome [10,11].

This study showed that some of the socio-economic conditions affect the weight of new born negatively. In this regard, mothers who resided in rural areas were more likely to deliver low birth weight babies. This finding is in agreement with study done in Tanzania and India [12, 13]. But This result is in contrast to a study done in Jimma zone, Ethiopia where the risk of delivering low birth weight babies was found to be significantly higher in those mothers who were residing in urban areas than those living in rural areas [14]. The difference might be due to inadequate rest and continuous hard working during pregnancy among mothers in rural area.

This study revealed that mothers who are illiterate and in lower income level were at higher risk to deliver LBW babies. Similarly, the study conducted in Nepal and Lahore showed that maternal education and per capita income of the family per month were found to be significantly associated with birth weight of the new born [13, 15]. The possible explanation and implications could be the low economic status of the mothers in the study area with increased costs of living might hinder to care pregnant mothers in terms of nutrition and health care. Education also influences people’s perceptions and dispositions towards different activities including health activities and behaviour such as proper maternal feeding practices and maternal health service utilization.

However, this study revealed no association between occupational status and LBW and lack of decision power on their resource utilization and LBW which is different from other previous study findings [13, 16]. This finding supports the previous study in Tanzania where there was no statistically significant difference among mothers’ occupations regarding LBW of their new-borns [12].

Pregnancy is a life threatening condition in a majority of developing countries, Its anomalous outcome reduces the life expectancy of new borns and their mothers. In this study, mothers who encountered pregnancy related health problems during current pregnancy were at higher risk to deliver low birth weight baby than mothers who didn’t. This result is similar with a study done in India that showed mothers with any health problem during pregnancy were two times more likely to give low birth weight babies [17].

The risk of low birth weight was higher among mothers who didn’t attend antenatal care for current pregnancy as compared to mothers who attended ANC. This is consistent with a study done in Nepal which showed as birth weight was significantly associated ANC service utilization [13].

Antenatal visits of the pregnant mothers are very important as they provide chances for monitoring the fetal wellbeing and allow timely intervention for feto-maternal protection including nutritional counseling that a mother might receive. Likewise, birth spacing had significant association with LBW. Mothers with birth spacing of 2 years and below were more likely to deliver low birth weight baby than mothers who delivered with birth interval of 2 or more years. This finding is in-line with a study done in India that showed birth interval of < 2 years were at higher risk to deliver LBW baby [17]. These findings were also consistent with similar study done in south western Ethiopia, Tanzania and Iran [12, 18, 19]. This could be due to the fact that short inter-pregnancy interval might result in inadequate replenishment of maternal nutrient stores depleted in the previous pregnancy and lead to reduced fetal growth.

We found that mothers BMI less than 18 kg/m^2^ and height less than 1.50 m were more likely to deliver low birth weight babies. This findings were consistent with a study conducted in India which revealed that low birth weights were significantly higher among mothers with height <145 cm and BMI <18.5 kg/m^2^ [17]. It is also consistent with studies done in Southwestern Ethiopia and Tanzania [12, 18].

It is also consistent with another similar study where BMI (<18 kg/m2) two times prone to deliver low birth weight babies [20]. The mean BMI <18 kg/m^2^ were significantly higher in mothers who had LBW babies compared to those who delivered NBW babies in another case control study in Iran [19]. This might be because of the fact that anthropometric measurements directly or indirectly measures nutritional status. In this case a BMI of less than 18 indicates the presence of under-nutrition that reveals chronic malnutrition among adults. Hence; maternal under-nutrition can hinder the growth and development of fetus in the uterus.

In this study maternal age at first birth, history of alcohol drink and number of pregnancies didn’t have significant associations with low birth weight. But mothers who had history of Khat chewing were statistically higher at risk to deliver LBW as compared to mothers who didn’t chew Khat.

The sources of drinking water affect the health of the people that use it. If toilet facilities, water sources and cooking environment are poor among mothers, it will expose them to various infections that leads to poor pregnancy outcomes. Various household environmental factors have been implicated in adverse pregnancy outcomes. The combustion product of solid fuel in developing countries can cause many adverse health effects in people. Majority of pregnant women in developing countries are heavily exposed to indoor air pollution which attributes to low birth weight [21]. This study showed that 63.6 % of mothers with LBW babies and 74 % of mothers with NBW babies were used firewood as cooking facility in the study area. The likelihood of giving low birth weight baby was significantly higher among mothers who were used firewood for cooking than electricity. Similarly; mothers who were used kerosene more likely to deliver LBW babies than electricity users. This result supports the study done in India, mothers who were used firewood and kerosene to cook were more likely to gave low birth weight than those who were used electricity [11]. It is also in agreement with another study conducted in India that shows infants were born in households using kerosene, coal and biomass experienced significantly higher odds of low birth weight [22,23]. The pathology due to biomass smoke exposure leads to respiratory tract infections, wheezing, chronic bronchitis and chronic obstructive pulmonary diseases. The main component of incomplete combustion of biomass, carbon monoxide combines with hemoglobin to form carboxyhaemoglobin with reduced delivery of oxygen to tissues and developing fetus. This leads to low birth weight babies and increases perinatal deaths [23, 24, 25].

In this study, mothers who didn’t have separate room for cooking were more likely to experience low birth weight babies. This could be due to maternal exposure during pregnant to smoky kitchens which is not separated from the dwelling room might result to inhale chemicals from biomass fuels which contribute for low birth weight and perinatal mortality [26, 27].

## Conclusion

The findings of this study showed that the presence of significant association between the socio-economic, maternal and household environmental factors and birth weight of the new-borns among mothers who gave birth in Bale zone hospitals.

From socio-economic factors; not having formal education, being a resident in rural area, maternal age less than 20 at current birth and having monthly income less than 26$ were identified as risk factors for low birth weight.

Absence of antenatal care follow-up, birth spacing of 2 years and below, short maternal stature, maternal BMI of less than 18Kg/m^2^, presence of pregnancy induced health problems and having history of Khat chewing were among maternal factors identified as positively associated with low birth weight.

Not having separate room for cooking, being firewood user, being kerosene user & being animal dung user as energy source for cooking, and lack of proper hand washing practice such as use of water only or water with ash rather than water with soap were among the household environmental conditions that increase the risk of low birth weight baby.

This study identified various socio economic, maternal and environmental risk factors for low birth weight. Therefore; prevention strategy for low birth weight in this area should be designed to tackle these multiple risk factors for low birth weight. Income generation means such as small scale enterprises should give due attention for mothers. In addition; mothers should be encouraged to use family planning method so as to maximize birth intervals between subsequent births.

Health professionals should screen and consulate pregnant mothers who are at risk of having infants with LBW and ensure that women have access to essential health information on the causes of low birth weight. Public education and awareness on how to carry on a healthy pregnancy. Likewise; women should be linked to the appropriate maternal health services including antenatal care and nutritional counseling services.

Community sensitization should do to improve household environmental conditions, where the pregnant women live and work. This should be in focus of promoting to have separate kitchen from living rooms and to use non-smoky energy sources for cooking such as electricity or to be away from such activities.

## Abbreviations

ANC: Antenatal care
AOR: Adjusted odds ratio
BMI: Body mass index
CI: Confidence interval
cm: Centimeter
DHS: Demography and Health Survey
HH: House hold
HIV: Human immuno deficiency virus
Kg: Kilo gram
g: Gram
IUGR: Intra uterine growth retardation
LBW: Low birth weight
MDG: Millennium Development Goal
FMOH: Federal ministry of health
NBW: Normal birth weight
OR: Odds ratio
UTI: Urinary tract infection
USD: United States Dollar
WHO: World Health Organization
